# Genetic Characterization of Highly Pathogenic Avian Influenza A(H5N1) Clade 2.3.4.4b, Antarctica, 2024

**DOI:** 10.3201/eid3108.250186

**Published:** 2025-08

**Authors:** Victor Neira, Naomi Ariyama, Pablo N. Castillo-Torres, Barbara Brito, Gabriela Muñoz, Karla B. Díaz Morales, Leonardo I. Almonacid, Benjamín Bennett-Laso, Barbara Berazay, Miloš Barták, Rafael Medina, Marcelo González-Aravena, Catalina Pardo-Roa

**Affiliations:** Universidad de Chile, Santiago, Chile (V. Neira, N. Ariyama, G. Muñoz, B. Bennett-Laso, B. Berazay); Pontificia Universidad Católica de Chile, Santiago (P.N. Castillo-Torres, K.B. Díaz Morales, L.I. Almonacid, C. Pardo-Roa); University of Technology Sydney, Sydney, New South Wales, Australia (B. Brito); Masaryk University, Brno, Czech Republic (M. Barták); Emory University Center of Excellence for Influenza Research and Response, Atlanta, Georgia, USA (R. Medina); Instituto Antártico Chileno, Punta Arenas, Chile (M. González-Aravena)

**Keywords:** avian influenza, highly pathogenic avian influenza, H5N1, HPAIV, genetic characterization, influenza, respiratory infections, viruses, zoonoses, Antarctica

## Abstract

In 2024, we sequenced highly pathogenic avian influenza virus A(H5N1) clade 2.3.4.4b genomes isolated from 5 brown skuas from James Ross Island, Antarctica. Phylogenetic analysis suggested the virus reached Antarctica through South America. Continued genetic surveillance will be critical to elucidate H5N1 virus transmission dynamics within Antarctica and surrounding areas.

Highly pathogenic avian influenza (HPAI) A(H5N1) has been causing a panzootic since its resurgence in 2021 (*1*). H5N1 clade 2.3.4.4b virus has spread among domestic and wild animals; multiple spillovers into distinct mammal species have occurred (*2*). This virus reached South America in late 2022, causing numerous mortality events in wild birds and mammals along the coasts of Peru, Chile, Argentina, Uruguay, and Brazil (*2*,*3*). By late 2023, the virus had extended its range to the South Atlantic and Antarctic Oceans; virus was detected on the Malvinas/Falkland Islands and Bird Island, South Georgia (*4*,*5*). Bird Island is a subantarctic island located near the Antarctic Peninsula, raising concerns about potential virus spread to previously unaffected ecosystems. 

In early 2024, H5N1 virus reached Antarctica; 11 cases were reported during the 2023–24 summer season (*6*). The virus primarily affected skuas seabirds but has also been detected in Adélie penguins, Antarctic fur seals, snowy sheathbills, kelp gulls, and southern elephant seals, according to the Scientific Committee on Antarctic Research (SCAR, https://scar.org/library-data/avian-flu#cases). We genetically characterized HPAI H5N1 virus samples collected on March 3, 2024, from brown skuas (*Stercorarius antarcticus*) on James Ross Island, located near the eastern side of the Antarctic Peninsula (latitude –63.7989S, longitude –57.8105W). The Faculty of Veterinary Sciences Ethics Committee, Universidad de Chile, approved the study (code no. 13–2022), which we registered with the Institutional Animal Care and Use Committee (code no. 22603-VET-UCH).

## The Study

We collected 6 pooled swab samples from dead brown skuas during a mass mortality event and confirmed the presence of H5N1 clade 2.3.4.4 virus in 5 birds by using a US National Veterinary Services Laboratories protocol (*6*). Those were the only positive samples from a Chilean Antarctic Institute/University of Chile surveillance program conducted during the Southern Hemisphere summer of 2023–24 (*6*). We performed full virus genome sequencing by using multisegment PCR and MinION nanopore sequencing (Oxford Nanopore Technologies, https://www.nanoporetech.com), as previously described (*7*). After obtaining consensus genomes, we checked sequences for quality and annotated them by using the Influenza Virus Sequence Annotation Tool (https://www.ncbi.nlm.nih.gov/genomes/FLU/annotation). We performed H5 clade classification by using the Subspecies Classification tool (Bacterial and Viral Bioinformatics Resource Center, https://www.bv-brc.org). We deposited sequences in GenBank (Appendix 1 Table).

We aligned sequences from the H5N1 samples with sequences from GISAID (https://www.gisaid.org) and GenBank by using MAFFT (*8*). We aligned 245 neuraminidase (NA), 259 hemagglutinin (HA), 229 polymerase basic 2, 256 nucleoprotein, 231 polymerase basic 1, 233 polymerase acidic, 254 nonstructural (NS), and 249 matrix gene sequences. We inferred time-divergent phylogenetic trees by using BEAST version 1.10.4 (*9*) and the Hasegawa-Kishino-Yano plus gamma distribution 4 substitution model, an uncorrelated relaxed clock with a lognormal distribution, and an exponential growth tree prior. We ran a Markov Chain Monte Carlo chain for 500 million generations, logging parameters every 50,000 iterations. We assessed the convergence of parameters by using Tracer version 1.7.2 (http://beast.community/tracer). The final trees had an effective sample size (ESS) of >200, except the trees for NS and polymerase acidic segments, which each had an ESS of <200; no trees had an ESS of <100. We visualized the trees and annotations by using iTol (*10*).

We obtained 6 influenza A virus genomes, all classified as HPAI H5N1 clade 2.3.4.4b, displaying minimal variation; we observed >99.9% identity in the HA genes and consistent results across other genomic segments. The closest HA sequence identified via BLAST (https://blast.ncbi.nlm.nih.gov) was A/Kelp Gull/South Georgia and the South Sandwich Islands/32/2023 (H5N1) virus (GenBank accession no. PQ113961.1), along with related sequences from the same outbreak. Similar findings were observed for all other segments.

Phylogenetic analysis of HA grouped the 6 sequences into a monophyletic cluster (Appendix 1 Figure 1), which was part of a larger clade of sequences previously reported from South Georgia and the South Sandwich Islands and derived directly from South America (*5*). Another subcluster contained sequences from King George Island (Antarctica), detected on December 25, 2024, suggesting a different introduction; however, those viruses were still related to the viruses from South America and subantarctic regions. We observed similar patterns across all other H5N1 segments (Appendix 1 Figures 2–8), indicating the virus reached Antarctica through local migrations and progressively spread across the region. The timeline suggests an initial introduction into southern South America, followed by spread across the South Atlantic Ocean to South Georgia and finally to the Antarctic Peninsula and the islands on its western and eastern coasts.

To identify key mutations, we analyzed all available H5N1 sequences from Antarctica and subantarctic regions, along with sequences from humans, dairy cows, seals, and chickens, by using FluSurver (http://flusurver.bii.a-star.edu.sg) and compared those against genome segments from the reference strain A/goose/Guangdong/1/96 (GenBank accession nos. AF144300–7). The polymerase basic 2 D701N mutation, associated with mammal adaptation, was detected in a virus from a subantarctic kelp gull, South Georgia A/Kelp_Gull/Harpon_Bay/133943/2023 (GISAID accession no. EPI_ISL_18592427). Mutations associated with mammal adaptations were absent in virus sequences obtained from skuas. However, we detected mutations associated with high-level resistance to amantadine (matrix, V27A) (*11*), antigenic drift (NA, I396M and N366I) (*12*), and virulence (NS, S48X and I205X) in the 5 sequences from skuas (*13*) (Appendix 2 Table).

The first limitation of our study is that it was based solely on sequences from 1 outbreak detected in 2024. However, other sequences from this region are not available, suggesting potential limitations in sequencing capacity or research efforts. To address those limitations, increased collaboration among research teams will be crucial to expand sequencing and elucidate virus spread in the region. Cost-effective technologies, such as nanopore sequencing, which enables whole-genome influenza A virus sequencing by using affordable equipment, should be prioritized to strengthen sequencing capabilities and data availability. Second, we only have sequences from brown skuas, which have been proposed to be the same species as south polar skuas (*Stercorarius maccormicki*) (*14*). Obtaining more H5N1 sequences from other species is essential to determine potential transmission pathways or virus adaptations that might occur across different hosts. Furthermore, SCAR data indicate the virus remained in Antarctica during the 2024–25 season, making it critical to study its dynamics and persistence in the region.

Antarctica has a plausible risk for H5N1 virus reassortment events, particularly those involving gene segments from strains from South America. The region’s animal populations, including penguins, skuas, gulls, and marine mammals, can act as mixing vessels for virus strains with distinct genetic backgrounds, promoting genetic exchange between viruses circulating in South America and other global regions. Reassortment in HA and NA genes, key determinants of virus fitness, host tropism, and transmissibility, is of particular concern. Those segments might undergo selective pressure from local host species, leading to adaptations that influence virus infectivity. For example, a H5N5 strain isolated from a chinstrap penguin, A/chinstrap_penguin/Antarctica/B04/2015 (H5N5) low pathogenicity avian influenza virus (AIV), showed phylogenetic links to AIVs from both North America and South America, suggesting potential reassortment events (*15*). In addition, the overlap of migratory bird routes with penguin breeding colonies creates a dynamic interface for AIV spillover and interspecies transmission, further supporting a role for Antarctica as a critical site for AIV reassortment and emergence of novel virus strains.

## Conclusions

We genetically characterized HPAI A(H5N1) clade 2.3.4.4b viruses found in skuas in Antarctica. That clade has also been detected farther south on the Antarctic Peninsula (according to SCAR); however, those sequences are not yet publicly available for analysis. Our findings indicate that continued genetic surveillance and collaborative efforts to expand sequencing across diverse species in Antarctica will be critical to elucidate transmission dynamics, host adaptation, and spread of HPAI H5N1 in Antarctica and surrounding areas.

Appendix 1 Additional information for genetic characterization of highly pathogenic avian influenza A(H5N1) clade 2.3.4.4b, Antarctica, 2024.

Appendix 2 Key mutations in highly pathogenic avian influenza A(H5N1) clade 2.3.4.4b, Antarctica, 2024.
