# Supplementary material for: Genetic Characterization of Highly Pathogenic Avian Influenza A(H5N1) Clade 2.3.4.4b, Antarctica, 2024
Source: Emerg Infect Dis. 2025 Aug;31(8):1618–20. doi: 10.3201/eid3108.250186 (PMC12309765; doi:10.3201/eid3108.250186)
Supplement: Appendix 1 — Additional information for genetic characterization of highly pathogenic avian influenza A(H5N1) clade 2.3.4.4b, Antarctica, 2024. [file 25-0186-Techapp-s1.pdf]

Article DOI: https://doi.org/10.3201/eid3108.250186

EID cannot ensure accessibility for supplementary materials supplied by authors. Readers who have difficulty accessing supplementary content should contact the authors for assistance.

Genetic Characterization of Highly Pathogenic Avian Influenza A(H5N1) Clade 2.3.4.4b, Antarctica, 2024

Appendix 1

Additional Methods

The samples were reextracted according to the protocol previously described (*3*, main text). Briefly, extraction was performed by using TRIzol lysis (15596018; Invitrogen, https://www.thermofisher.com) plus E.Z.N.A Viral RNA Kit (R6874; Omega Biotek, https://www.omegabiotek.com). Then, the whole genome was amplified, and PCR products were purified by using SPRISelect Beads (B2338; Beckman Coulter, https://beckman.com). High-quality samples were sequenced by using the nanopore technology (ONT) platform with the Native Barcoding Kit (SQK-NBD114.96; Oxford Nanopore Technologies, https://www.nanoporetech.com) according to the manufacturer's instructions for R.10 flow cells. The genomes were assembled by referencing the genome segments of A/*Falco_rusticolus*/EdoMex/CPA-19638–22/2022(H5N1) (GenBank accession nos. OP691321–8).

Appendix 1 Table. GenBank accession numbers*

				Influenza virus gene segments							
Date	Sample identification no.	Subtype	Coverage†	PB2	PB1	PA	HA	NP	NA	M	NS
03–03–24	INACH-UC-UCHILE-SKU1	H5N1	24.077	PQ304442	PQ304438	PQ304437	PQ304440	PQ304443	PQ318382	PQ304441	PQ304439
03–03–24	INACH-UC- UCHILE -SKU2	H5N1	18.192	PQ304431	PQ304434	PQ304436	PQ304435	PQ304433	PQ318403	PQ304430	PQ304432
03–03–24	INACH-UC- UCHILE -SKU3	H5N1	8.834	PQ304423	PQ304426	PQ304428	PQ304425	PQ304429	PQ318398	PQ304424	PQ304427
03–03–24	INACH-UC- UCHILE -SKU4	H5N1	18.477	PQ304582	PQ304586	PQ304587	PQ304584	PQ304583	PQ318371	PQ304588	PQ304585
03–03–24	INACH-UC- UCHILE -SKU5	H5N1	16.184	PQ304483	PQ304486	PQ304487	PQ304488	PQ304484	PQ318388	PQ304482	PQ304485
03–03–24	INACH-UC- UCHILE -SKU5L	H5N1	17.681	PQ304563	PQ304565	PQ304564	PQ304566	PQ304567	PQ318393	PQ304568	PQ304569

*HA, hemagglutinin; M, matrix; NA, neuraminidase; NP, nucleoprotein; NS, nonstructural; PA, polymerase acidic; PB1, polymerase basic 1; PB2, polymerase basic 2.
†Sequencing depth or coverage, number of reads obtained.

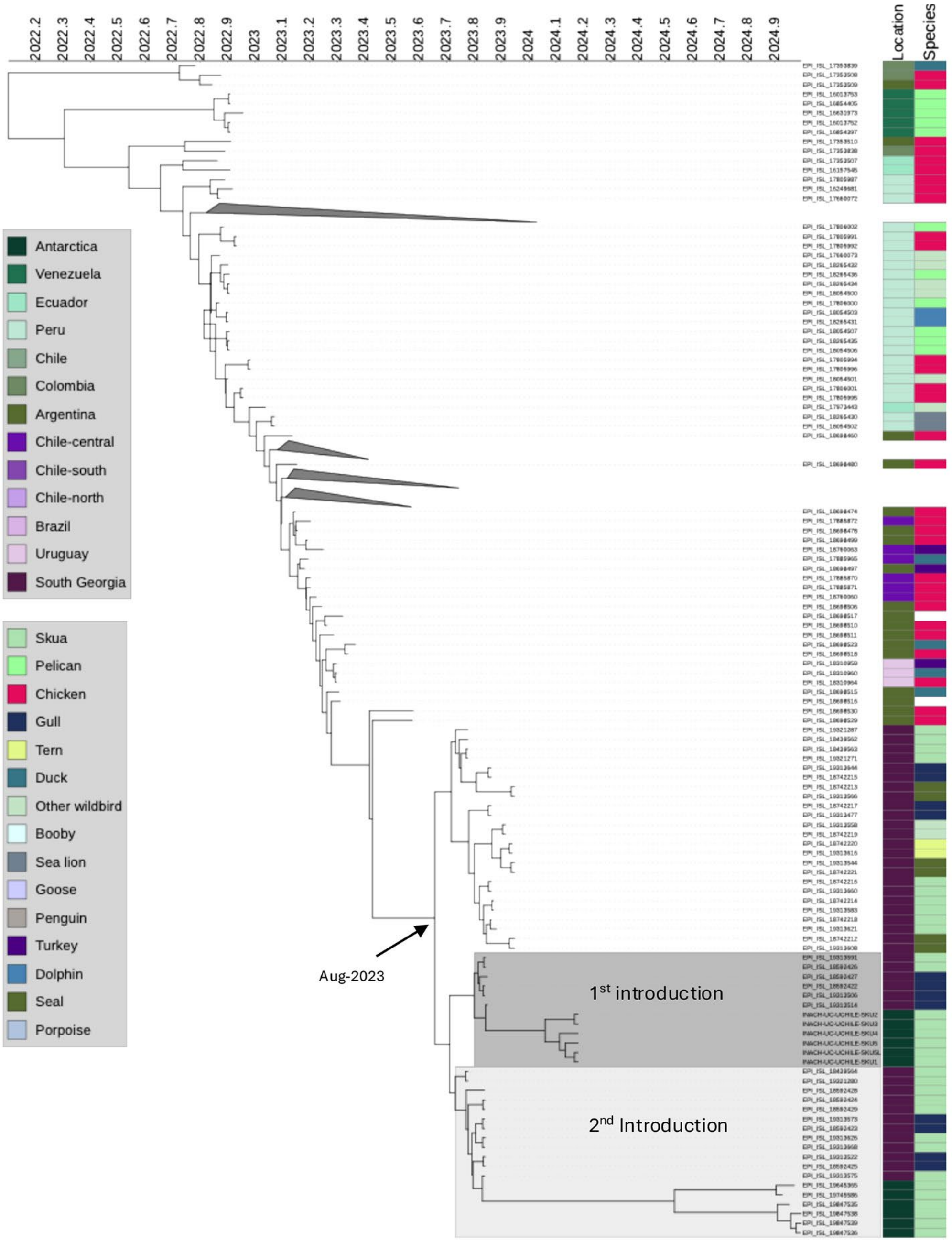

Appendix 1 Figure 1. Phylogenetic analysis of H5 clade 2.3.4.4b influenza viruses identified in Antarctica, 2024. Maximum clade credibility tree depicting time to most recent common ancestor estimates, generated by using a log-normal distribution and exponential growth models. Tree was reconstructed by using H5 gene sequences of strains sequenced from South America, South Georgia Islands, and Antarctica. The 6 sequences from this study were grouped into a monophyletic cluster during the first introduction. Another subcluster contained sequences from King George Island (Antarctica), detected on December 25, 2024 during the second introduction (at bottom of tree; EPI_ISL_19847536, EPI_ISL_19847539, EPI_ISL_19847538, EPI_ISL_19847535, EPI_ISL_19745586, EPI_ISL_19645365). Scale bar indicates nucleotide substitutions per site.

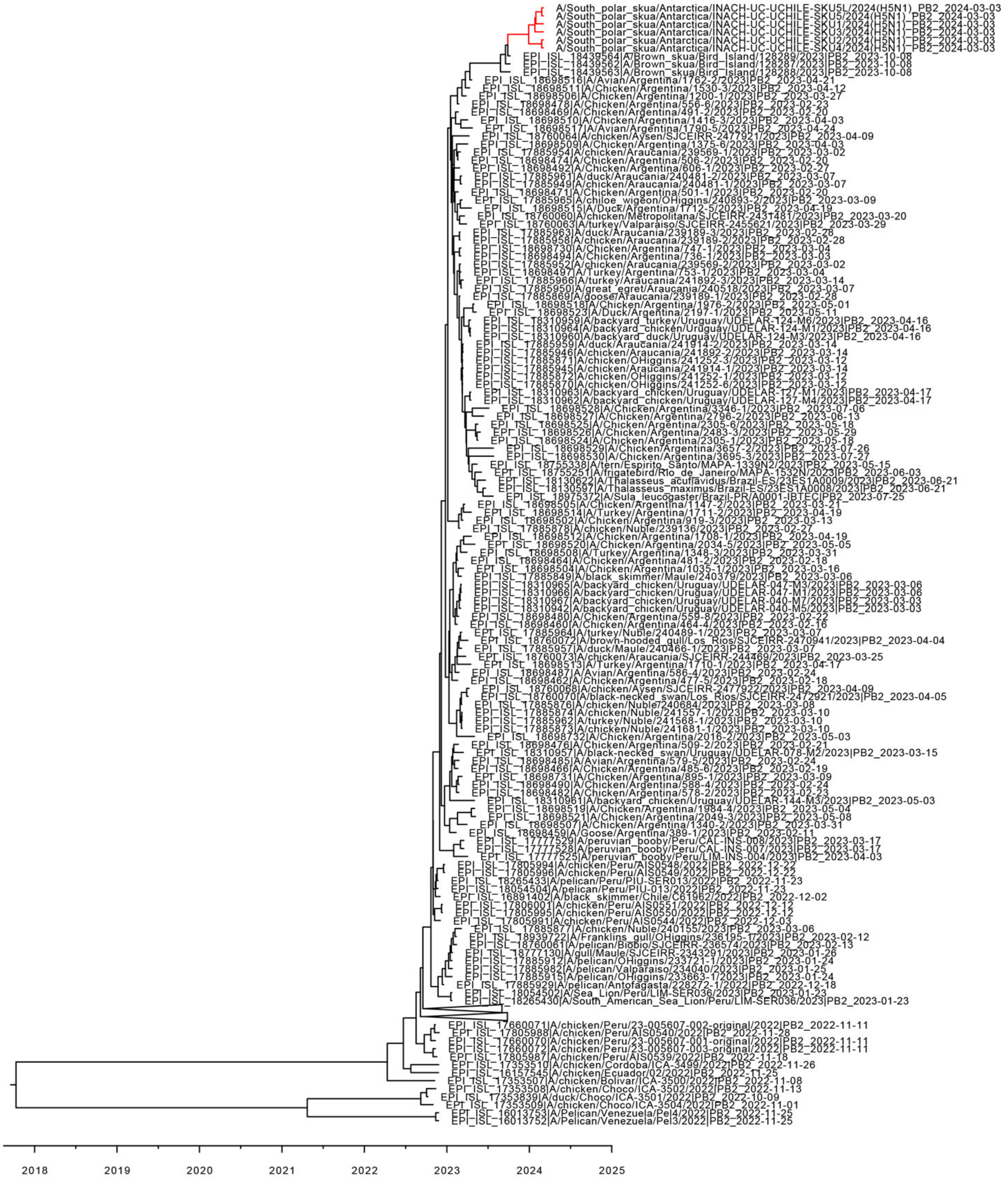

Appendix 1 Figure 2. Time-scaled maximum clade credibility tree for polymerase basic 2 genes of avian influenza virus. Red branches indicate sequences from this study.

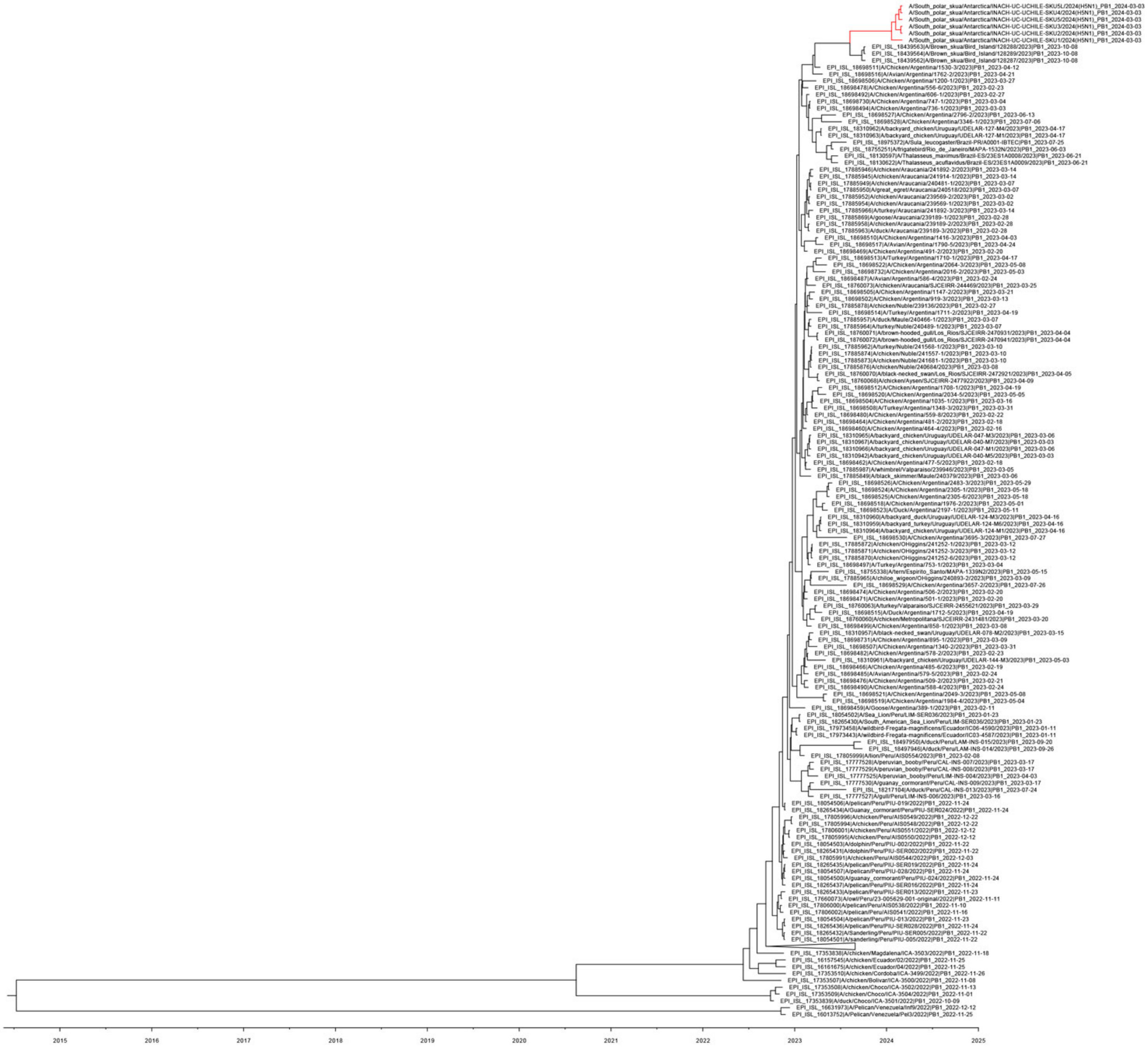

Appendix 1 Figure 3. Time-scaled maximum clade credibility tree for polymerase basic 1 genes of avian influenza virus. Red branches indicate sequences from this study.

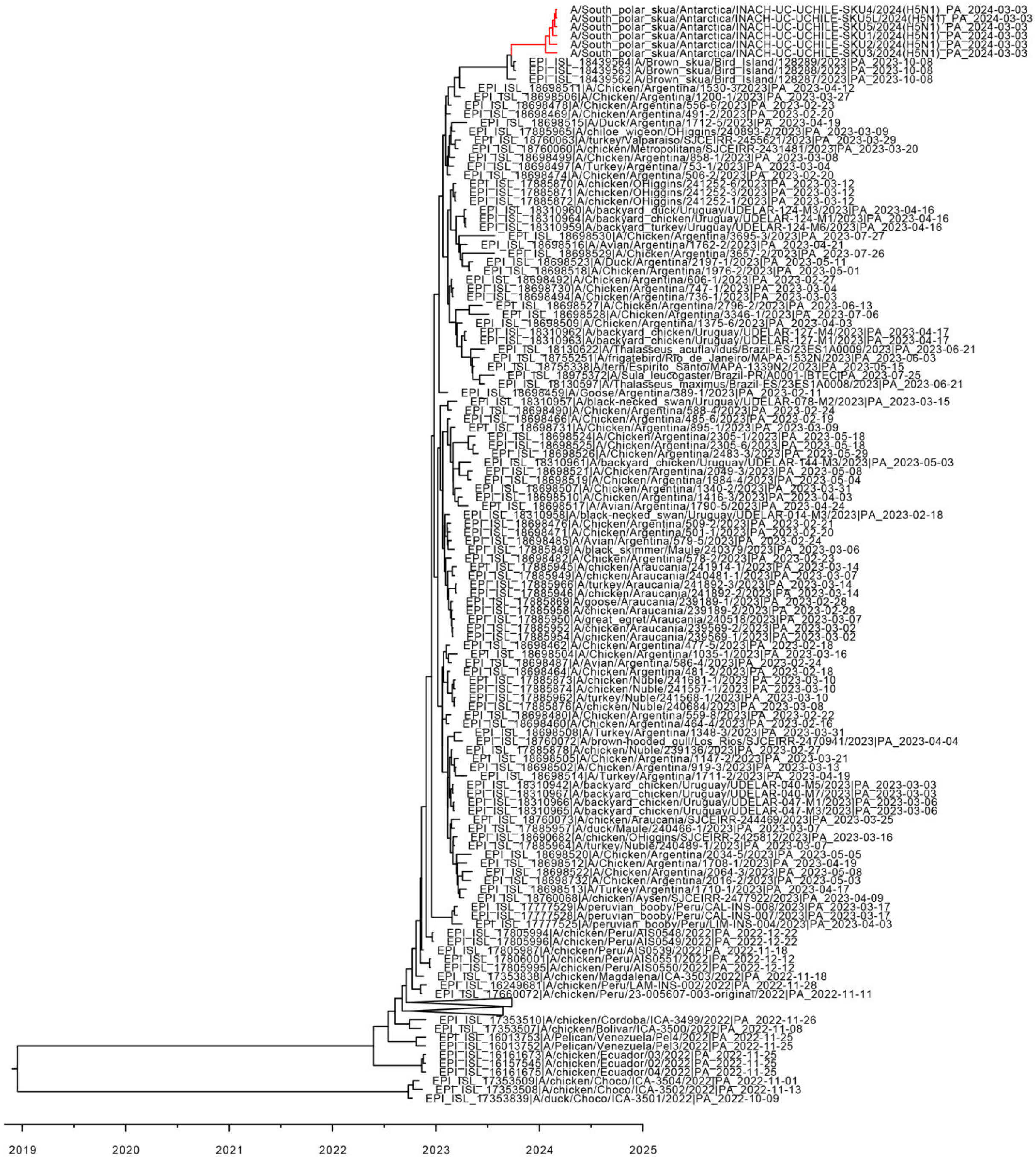

Appendix 1 Figure 4. Time-scaled maximum clade credibility tree for polymerase acidic genes of avian influenza virus. Red branches indicate sequences from this study.

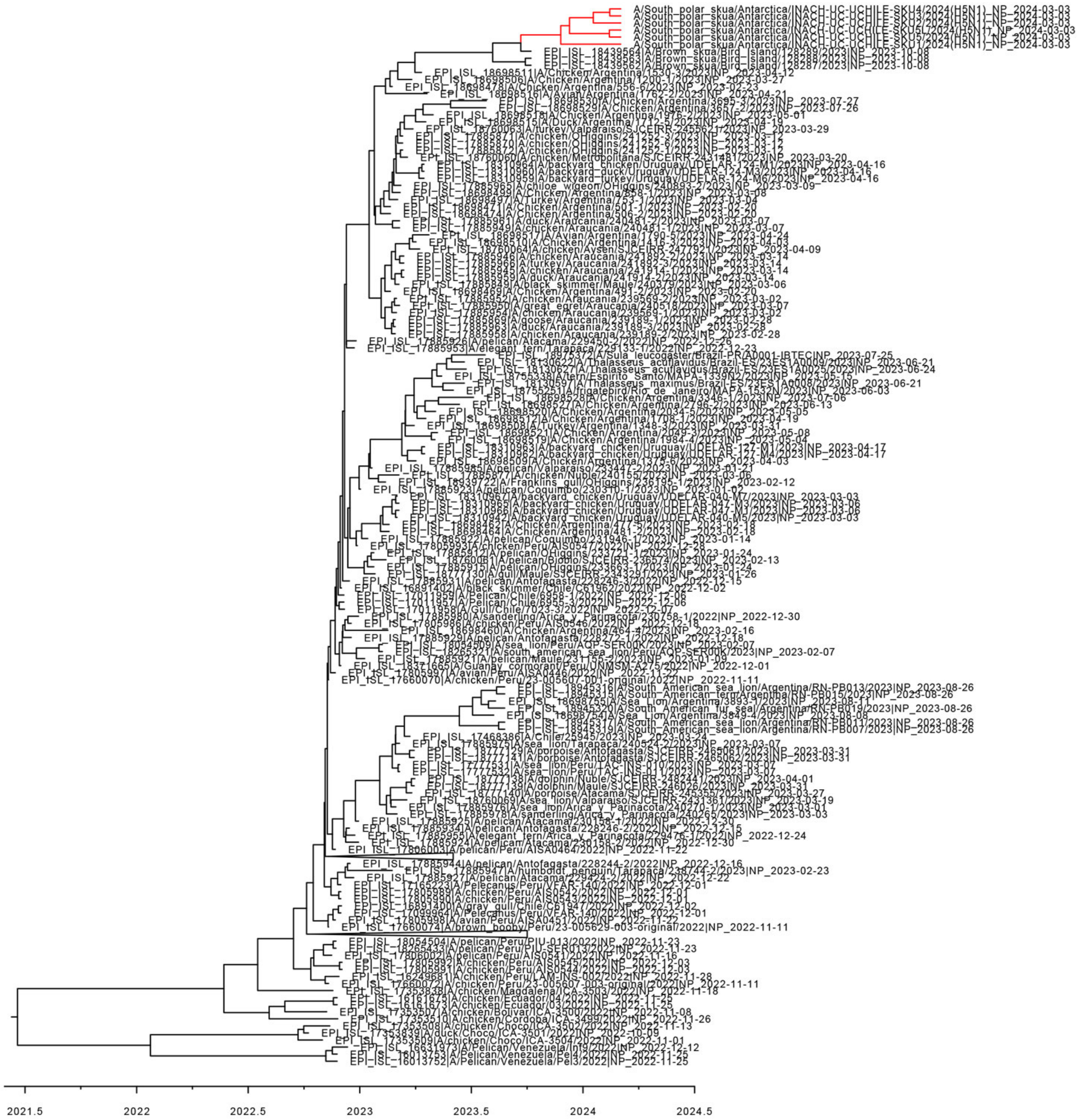

Appendix 1 Figure 5. Time-scaled maximum clade credibility tree for nucleoprotein genes of avian influenza virus. Red branches indicate sequences from this study.

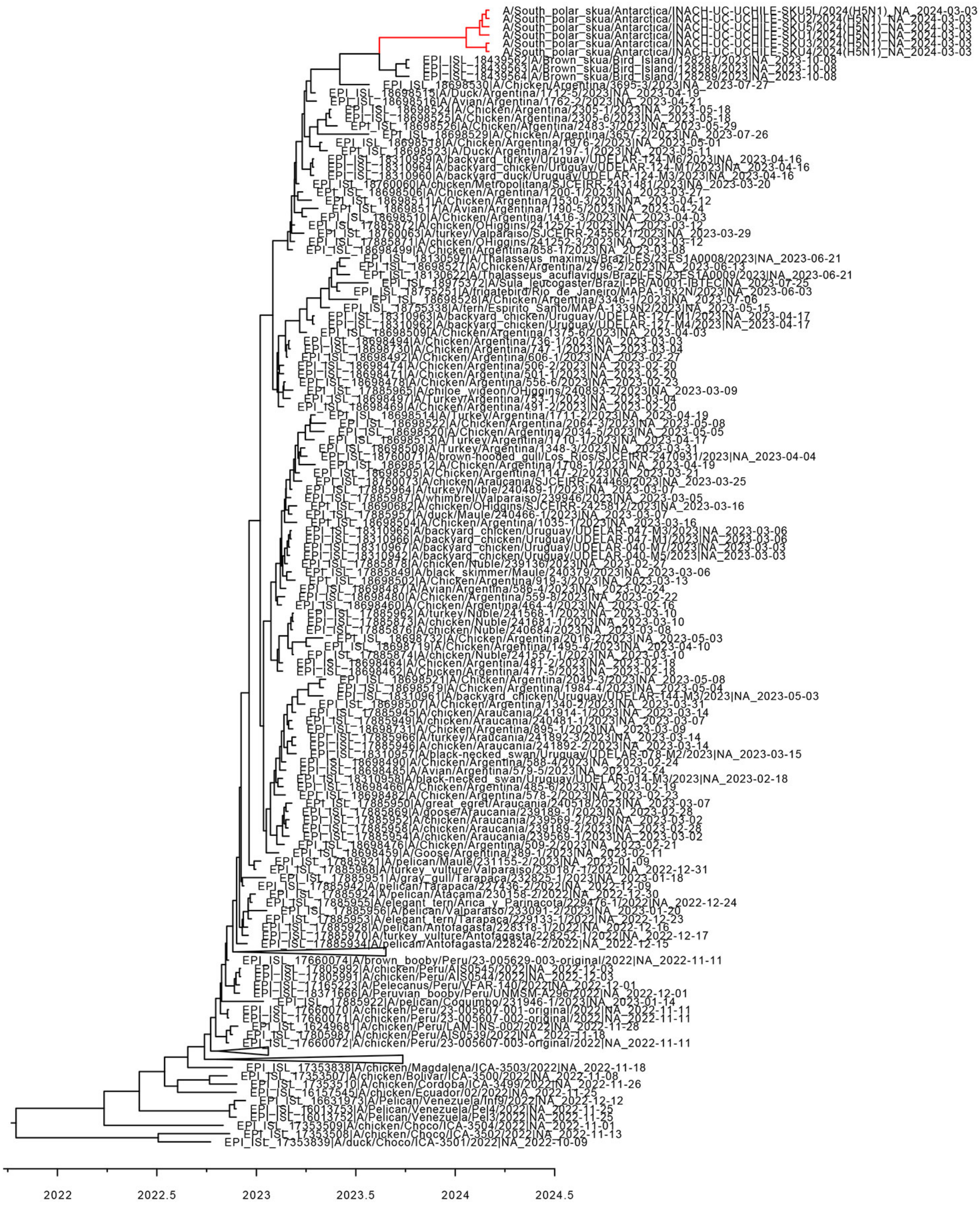

Appendix 1 Figure 6. Time-scaled maximum clade credibility tree for neuraminidase genes of avian influenza virus. Red branches indicate sequences from this study.

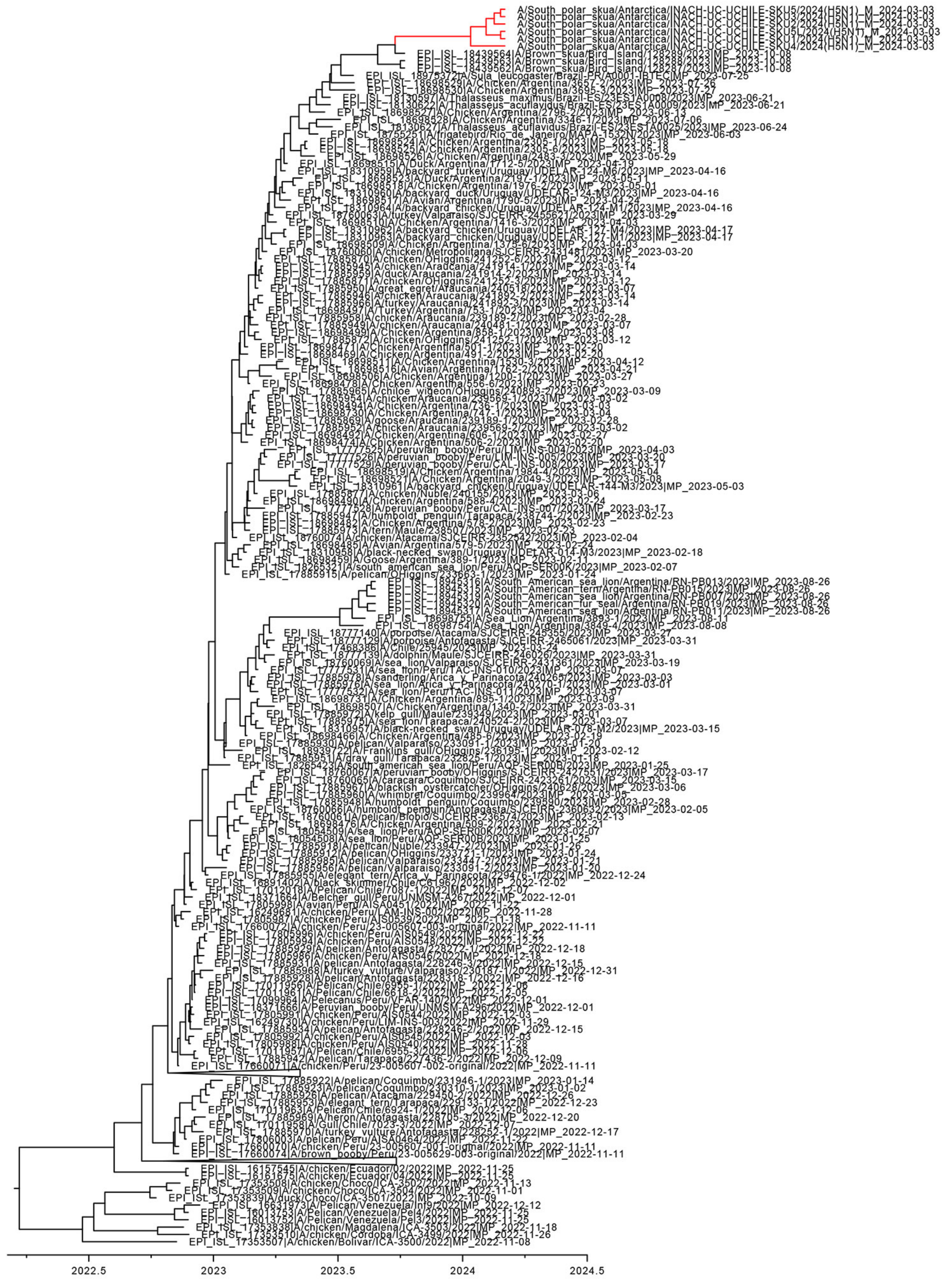

Appendix 1 Figure 7. Time-scaled maximum clade credibility tree for matrix genes of avian influenza virus. Red branches indicate sequences from this study.

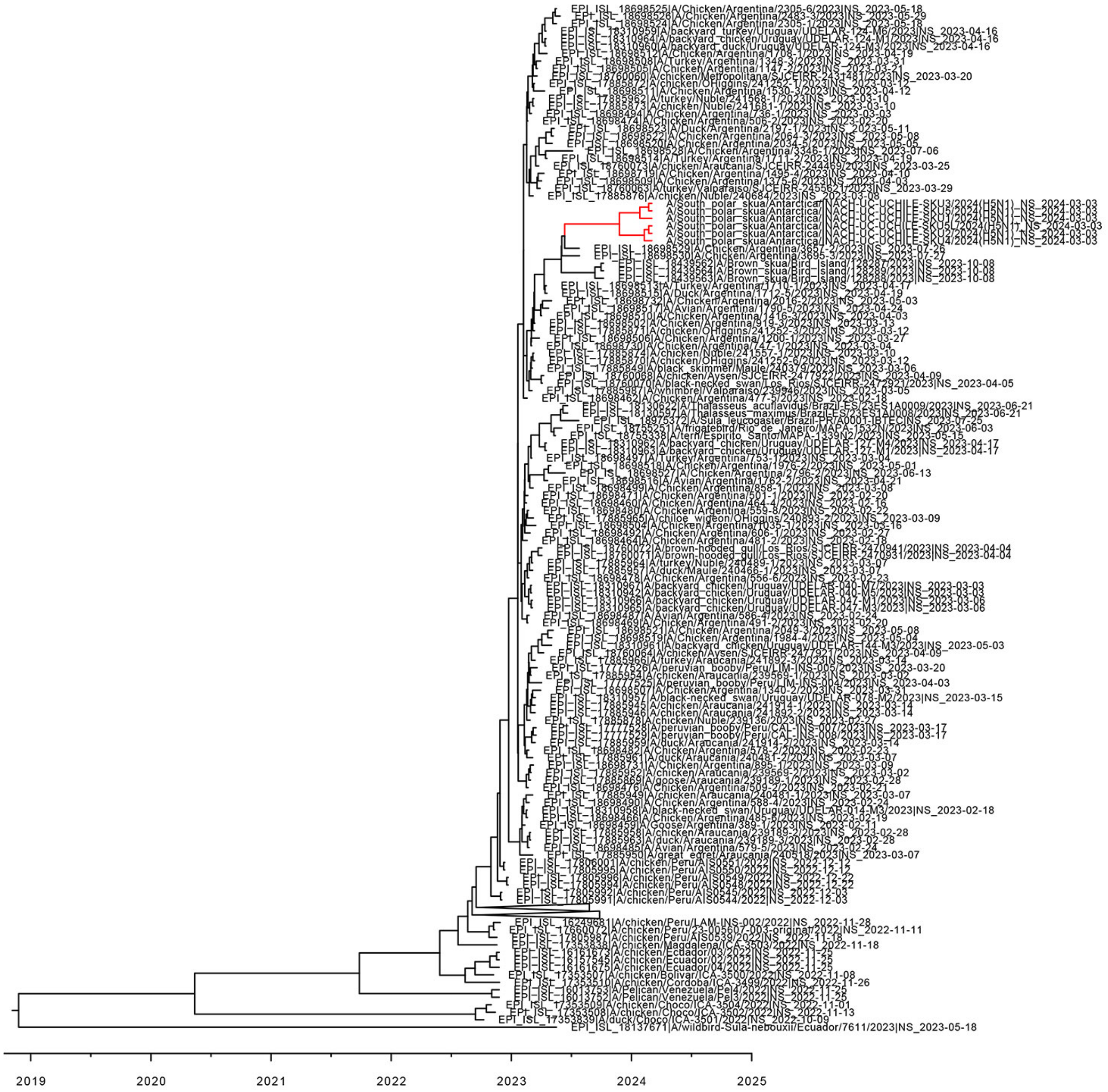

Appendix 1 Figure 8. Time-scaled maximum clade credibility tree for nonstructural genes of avian influenza virus. Red branches indicate sequences from this study.
